# High Mitophagy and Low Glycolysis Predict Better Clinical Outcomes in Acute Myeloid Leukemias

**DOI:** 10.3390/ijms252111527

**Published:** 2024-10-27

**Authors:** Amreen Salwa, Alessandra Ferraresi, Letizia Vallino, Chinmay Maheshwari, Riccardo Moia, Gianluca Gaidano, Ciro Isidoro

**Affiliations:** 1 Laboratory of Molecular Pathology, Department of Health Sciences, Università del Piemonte Orientale, Via P. Solaroli 17, 28100 Novara, Italy; salwa.amreen@uniupo.it (A.S.); alessandra.ferraresi@med.uniupo.it (A.F.); letizia.vallino@uniupo.it (L.V.); chinmay.maheshwari@uniupo.it (C.M.); 2 Division of Hematology, Department of Translational Medicine, Università del Piemonte Orientale, Via P. Solaroli 17, 28100 Novara, Italy; riccardo.moia@uniupo.it

**Keywords:** personalized medicine, leukemia, autophagy, mitochondria, Warburg effect, overall survival, TCGA, bioinformatics

## Abstract

Acute myeloid leukemia (AML) emerges as one of the most common and fatal leukemias. Treatment of the disease remains highly challenging owing to profound metabolic rewiring mechanisms that confer plasticity to AML cells, ultimately resulting in therapy resistance. Autophagy, a highly conserved lysosomal-driven catabolic process devoted to macromolecular turnover, displays a dichotomous role in AML by suppressing or promoting disease development and progression. Glycolytic metabolism represents a pivotal strategy for AML cells to sustain increasing energy needs related to uncontrolled growth during disease progression. In this study, we tested the hypothesis that a high glycolytic rate and low autophagy flux could represent an advantage for AML cell proliferation and thus be detrimental for patient’s prognosis, and vice versa. TCGA in silico analysis of the AML cohort shows that the high expression of *MAP1LC3B* (along with that of *BECN1* and with low expression of *p62*/*SQSTM1*) and the high expression of *BNIP3* (along with that of *PRKN* and of *MAP1LC3B*), which together are indicative of increased autophagy and mitophagy, correlate with better prognosis. On the other hand, the high expression of glycolytic markers *HK2*, *PFKM*, and *PKM* correlates with poor prognosis. Most importantly, the association of a low expression of glycolytic markers with a high expression of autophagy–mitophagy markers conferred the longest overall survival for AML patients. Transcriptomic analysis showed that this combined signature correlates with the downregulation of a subset of genes required for the differentiation of myeloid cells, lactate/pyruvate transporters, and cell cycle progression, in parallel with the upregulation of genes involved in autophagy/lysosomal trafficking and proteolysis, anti-tumor responses like beta-interferon production, and positive regulation of programmed cell death. Taken together, our data support the view that enhanced autophagy-mitophagy flux together with low glycolytic rate predisposes AML patients to a better clinical outcome, suggesting that autophagy inducers and glucose restrictors may hold potential as adjuvant therapeutics for improving AML management.

## 1. Introduction

Acute myeloid leukemia (AML) is an aggressive hematologic neoplasm characterized by an intense proliferation of immature myeloblasts in the bone marrow and poor prognosis. Induction and consolidation chemotherapy has improved the remission rates and five-year survival, yet about 60–80% of patients eventually relapse due to the onset of chemoresistance [1,2]. Recently, novel molecular therapies targeting metabolic and cell death pathways have been introduced [3]. Among the multiple factors driving drug resistance, glucose metabolic rewiring and mitochondrial dysfunction appear crucial in AML [3,4,5].

The Warburg effect, or aerobic glycolysis, is a common metabolic alteration in cancer cells and consists of the defective mitochondrial oxidation of pyruvate that is instead converted into lactate [6].

One key component in this metabolic reprogramming is the upregulation of mitochondrial hexokinase 2 (HK2), an enzyme critical for glycolysis initiation. In chemoresistant AML cells, internal tandem duplication mutation in the *FLT3* (Fms-like tyrosine kinase 3) gene leads to the hyper-expression of HK2 and increased glycolytic flux, which makes the leukemic cells sensitive to glycolysis inhibitors [7].

In challenging conditions such as nutrient and glucose deprivation, oxidative stress, and hypoxia, cells activate autophagy, a cytoprotective mechanism aimed at maintaining macromolecular and organelle homeostasis and preventing oncogenesis. Autophagy is regulated by key signaling cascades that include mTOR, AMPK, and BECLIN1-PI3KC3 pathways [8]. Here, the HK2-mTOR interaction functions as a molecular switch between glycolysis and autophagy to meet the bioenergetic demands during glucose starvation [9].

Dysregulated autophagy correlates with genomic instability and an increased mutational burden, which plays a role in the onset of various cancers, including AML [10,11]. Autophagy has conflicting roles in cancers, particularly in AML, with regard to different stages of cancer development. While it primarily regulates hematopoiesis and safeguards the blast cell populations from malignant transformation, the inhibition of autophagy sensitizes AML cells to chemotherapeutics [12,13,14]. The bone marrow microenvironment frequently experiences hypoxia [15], which is crucial in the regulation of cancer stem cell maintenance [16] and the selection of chemoresistant clones [17]. Within the hypoxic bone marrow microenvironment, HIF-1α actively induces autophagy in stem cells [18].

Hypoxia-induced autophagy represents an adaptive mechanism that increases chemoresistance and survival of AML blasts through the selective removal of dysfunctional mitochondria (also known as mitophagy) [19]. Consistently, it has been shown that the pharmacologic inhibition of mitophagy compromises the survival of leukemic stem cells [3,12].

Here, we interrogated the TCGA database to address the translational relevance of autophagy–mitophagy and of glycolysis–Warburg effect biomarkers in AML.

From an in silico analysis, we found that a high expression of *MAP1LC3B* (along with that of *BECN1* and with low expression of *p62*/*SQSTM1*) and the high expression of *BNIP3* (along with that of *PRKN* and of *MAP1LC3B*), which together are indicative of increased autophagy and mitophagy, correlate with a better prognosis in AML patients. On the other hand, the high expression of *PKM*, *PFKM*, and *HK2*, which code for enzymes of the glycolytic pathway (pyruvate kinase M1/2, phosphofructokinase, and hexokinase-2, respectively), correlates with poor prognosis in AML patients. Most importantly, the association of a low expression of glycolytic markers with a high expression of autophagy-mitophagy markers conferred the longest overall survival. Consistently, the patients categorized as the favorable-risk group (based on ELN 2008 risk classification) showed the lowest mRNA expression of *PFKM*.

Taken together, these data strongly support the contention that high autophagy–mitophagy flux and a low glycolytic rate are beneficial for AML patients, suggesting the usefulness of adjuvant therapeutics that stimulate autophagy (such as caloric restriction mimetics), meanwhile inhibiting (or lowering) glycolysis (as for instance, with glucose restriction mimetics and glycolytic enzyme inhibitors).

## 2. Results

### 2.1. Oncoprint Defines the Somatic Mutations of Mitophagy and Glycolysis Biomarkers in Acute Myeloid Leukemia (AML) Patients

**Figure 1:**
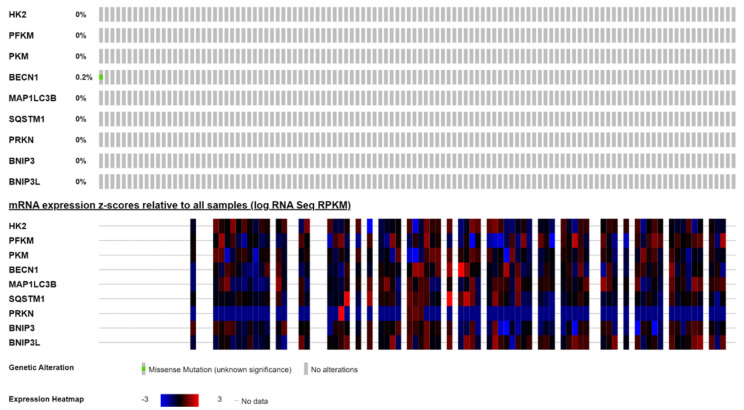
Oncoprint reporting copy number variations and expression profile. The oncoprint shows the genetic alterations (upper part) and mRNA expression levels in the AML patient datasets (TCGA, OHSU, Nature 2018). [Note: mRNA expression profiles of the above genes are available only for 405 out of 622 AML patients].

We monitored the copy number alterations and mRNA expression of selected markers involved in glycolysis (*HK2*, *PFKM*, *PKM*), autophagy (*BECN1*, *MAP1LC3B*, *SQSTM1*), and mitophagy (*PRKN*, *BNIP3*, *BNIP3L*) by interrogating the AML dataset (OHSU, Nature 2018) from the TCGA bioportal. As shown in the upper panel of Figure 1, except for one patient who displayed a missense mutation in *BECN1*, no alterations in the other selected genes were recorded. The heatmap in the bottom part of Figure 1 shows the heterogenic expression patterns of the selected glycolysis and autophagy–mitophagy markers.

Next, we correlated *PFKM* and autophagy–mitophagy markers (*MAP1LC3B* and *BNIP3*) with the most common genetic alterations in AML patients among which we chose *NPM1* and *FLT3*, accounting for 22% and 28% of the TCGA cohort, respectively. Because of the small number of patients, no statistically significant correlations were found (data shown as a Venn diagram in Supplementary Figure S1).

Further, we assessed the relationship between the glycolytic/autophagy–mitophagy markers with ELN 2008 risk classification (a gold standard in clinical practice) that includes favorable, intermediate, and adverse risk stratification groups. It was found that patients in the favorable-risk group had a low *PFKM* mRNA expression compared to the intermediate- and adverse-risk groups, while no significant changes were found for *MAP1LC3B* and *BNIP3* mRNA expression (Supplementary Figure S2).

### 2.2. High Expression of Glycolytic Markers Predicts Worse Clinical Outcomes in AML Patients

**Figure 2:**
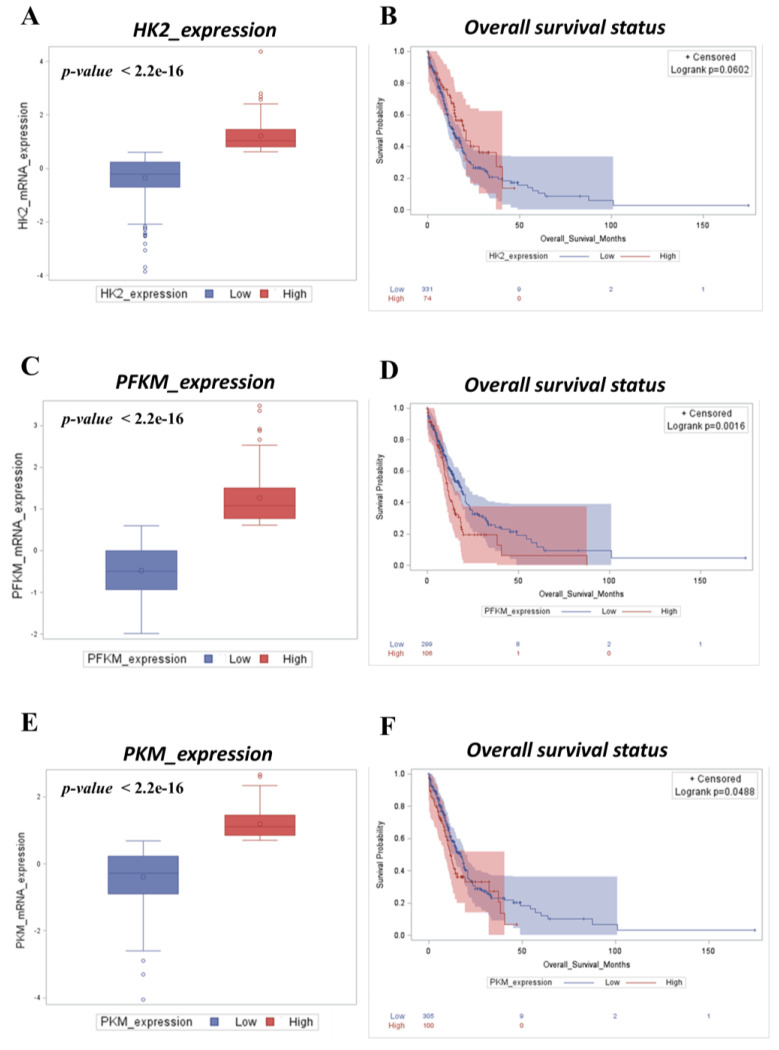
High expression of *HK2*, *PFKM*, and *PKM* is associated with poor prognosis in AML patients. (**A**,**C**,**E**) Box plots showing the distribution of *HK2* (**A**), *PFKM* (**C**), and *PKM*; (**E**) mRNA expression levels in AML patients (high vs. low). (**B**,**D**,**F**) Kaplan–Meier curves depicting the overall survival rate of AML patients, respectively, based on differential *HK2* (**B**), *PFKM* (**D**), and *PKM* (**F**) mRNA expression levels (low vs. high).

We determined the prognostic value of the glycolytic markers *HK2* (hexokinase-2), *PFKM* (phosphofructokinase), and *PKM* (pyruvate kinase M1/2) in the AML patient cohort (Figure 2). We found that patients bearing a tumor characterized by a high mRNA expression of the glycolytic genes were associated with an overall survival shorter than that of patients with a low expression of these genes. In particular, the stratification based on *PFKM* expression (Figure 2C,D) displayed the highest statistical significance (p = 0.0016), followed by *PKM* (Figure 2E,F; *p* = 0.0488) and *HK2*, slightly not significant (Figure 2A,B; *p* = 0.0602), respectively. For this reason, we selected *PFKM* to perform the subsequent transcriptomic analysis.

**Figure 3:**
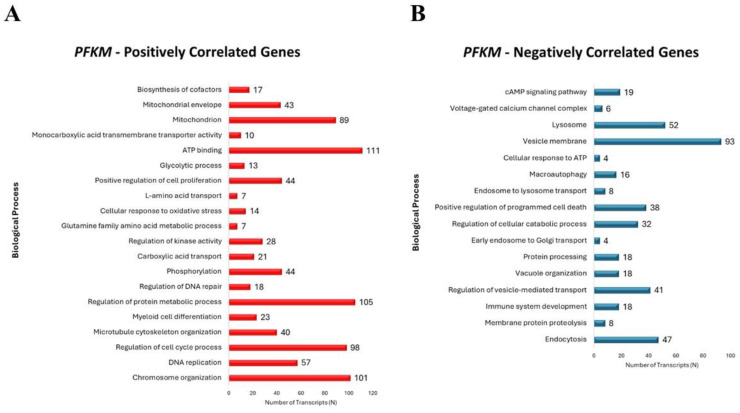
*PFKM* positively correlates with genes regulating cell cycle, myeloid cell proliferation, and glycolysis, whereas it negatively correlates with genes belonging to autophagy, proteolysis, and apoptotic cell death in AML patients. Bar graphs showing the Gene Ontology analysis reporting *PFKM*-positively (**A**) and negatively (**B**) correlated genes in AML patients, respectively.

To dissect the biological processes involved in the differential clinical outcomes described above, we performed an in silico transcriptomic analysis by retrieving *PFKM*-differentially expressed genes from the AML dataset of TCGA. The co-expression analysis reported in Figure 3A shows that *PFKM* positively correlates with transcripts involved in survival and cell proliferation, glycolysis, mitochondrial metabolism and homeostasis, ATP binding, and myeloid cell differentiation. On the other hand, Figure 3B shows that *PFKM*-negatively correlated transcripts are enriched in lysosomal genes, autophagy and catabolic processes, apoptosis regulation, and the cAMP signaling pathway. Taken together, these data indicate that a low expression of glycolytic genes predisposes to better clinical outcomes, also suggesting that *PFKM* may represent a valid prognostic marker for AML. This supports the above finding showing that the expression of *PFKM* is lower in patients in the favorable-risk groups compared to the intermediate- and high-risk groups.

### 2.3. Enhanced Autophagy–Mitophagy Associates with a Better Prognosis for AML Patients

**Figure 4:**
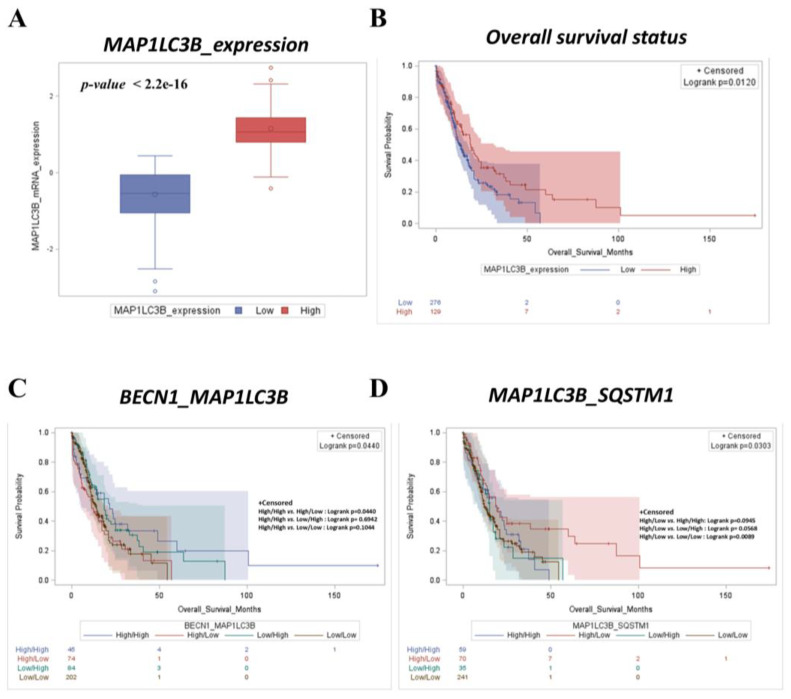
High *MAP1LC3B* expression, along with high *BECN1* and low *SQSTM1* expression is significantly associated with good prognosis in AML patients. (**A**) Box plot showing distribution of mRNA expression levels of *MAP1LC3B* (high vs. low). (**B**) Kaplan–Meier curve depicting overall survival rate of AML patients based on *MAP1LC3B* mRNA expression (low vs. high). (**C**,**D**) Kaplan–Meier curves representing overall survival status of AML patients stratified based on differential expression of *BECN1*/*MAP1LC3B* (**C**) and *MAP1LC3B*/*SQSTM1* (**D**) expression (high/high, high/low, low/high, and low/low groups, respectively).

The above results show that *PFKM* negatively correlates with genes belonging to the cellular catabolic process. This observation led us to hypothesize that the oncogenic role of glycolysis associated with bad prognosis in AML patients could be related to the downregulation of autophagy–mitophagy pathways. First, we tested the prognostic roles of *BECN1*, *SQSTM1*, and *MAP1LC3B* in the AML dataset. Although it did not reach statistical significance, the trend observed in Supplementary Figure S3 shows that the patients with a high expression of *BECN1* (*p* = 0.4582) and low expression of *SQSTM1* (*p* = 0.6789) displayed longer survival than their counterparts. More relevant, the stratification based on *MAP1LC3B* expression (Figure 4A,B) shows that the patients with a tumor expressing high *MAP1LC3B* survive longer than low-expressers (*p* = 0.0120). Consistently, the multiple-variable survival analysis reported in Figure 4C,D depicts that AML patients with high *BECN1*/high *MAP1LC3B* and high *MAP1LC3B*/low *SQSTM1* expression profiles (indicative of active autophagy) display better prognosis (*p* = 0.0440 vs. *p* = 0.0303, respectively).

**Figure 5:**
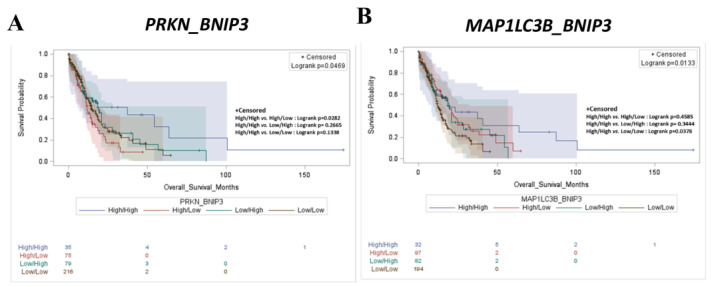
Enhanced expression of *BNIP3* together with high *PRKN* and high *MAP1LC3B* expression significantly correlates with longer overall survival in AML patients. (**A**,**B**) Kaplan–Meier plots representing overall survival status of AML patients stratified based on differential expression of *PRKN*/*BNIP3* (**A**) and *MAP1LC3B*/*BNIP3* (**B**) mRNA expression (high/high, high/low, low/high, and low/low groups, respectively).

Next, we performed the same survival analysis for mitophagy markers. We assessed the prognostic roles of *PRKN* (Parkin RBR E3 ubiquitin protein ligase), *BNIP3* (BCL2 interacting protein 3), and *BNIP3L* (BCL2 interacting protein 3-like). Supplementary Figure S4 shows that patients with a high mRNA expression of these mitophagy markers exhibit a longer survival rate (*p* = 0.5241, *p* = 0.0726, and *p* = 0.5068, respectively) compared to that of low-expressors, although these correlations were not statistically significant. More interestingly, statistical significance was reached in the correlation analysis showing that high *PRKN*/*BNIP3* and high *MAP1LC3B*/*BNIP3* (collectively indicating active mitophagy) signatures predict a better prognosis compared to their counterparts, where these genes are expressed at a low level (Figure 5; *p* = 0.0469 and *p* = 0.0133, respectively). Overall, these results indicate that high autophagy–mitophagy results in improved clinical outcomes, highlighting the fact that the activation of this axis predisposes to a milder disease.

### 2.4. AML Patients with Low Glycolysis and Enhanced Autophagy–Mitophagy Display Better Prognosis

**Figure 6:**
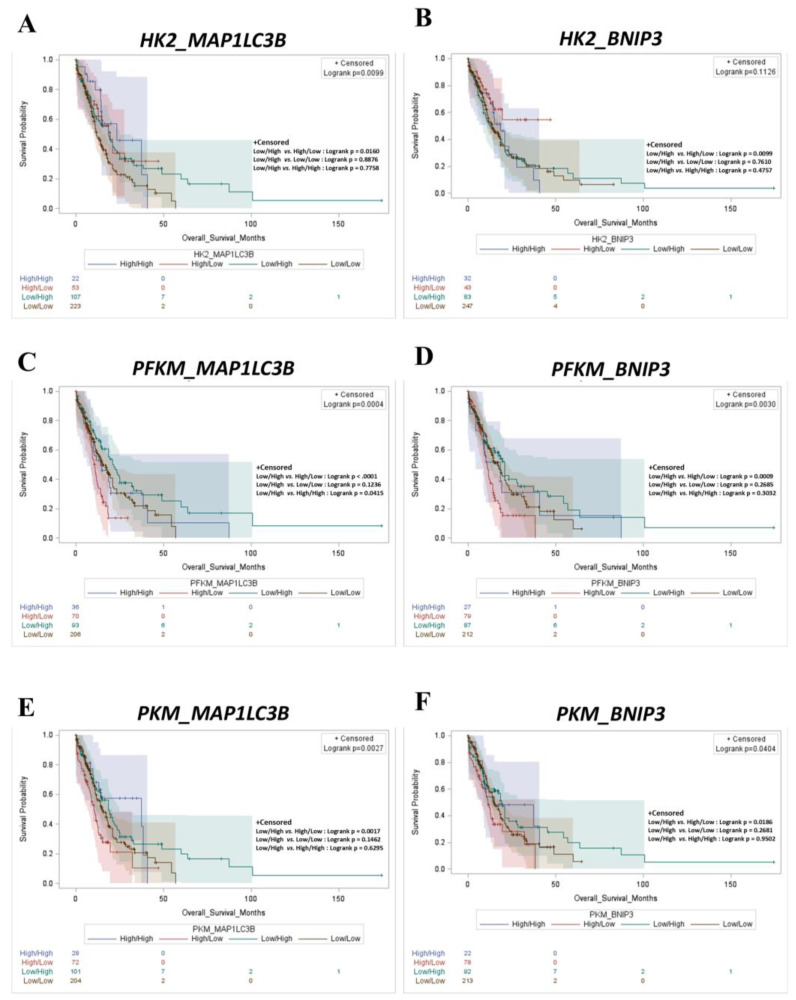
Low expression of glycolytic markers (*HK2*, *PKM*, and *PFKM*) with high *MAP1LC3B* or high *BNIP3* expression predicts longer overall survival in AML patients. Kaplan–Meier plots representing overall survival status of AML patients stratified based on differential expression of *HK2*/*MAP1LC3B* (**A**), *HK2*/*BNIP3* (**B**), *PFKM*/*MAP1LC3B* (**C**), *PFKM*/*BNIP3* (**D**), *PKM*/*MAP1LC3B* (**E**), and *PFKM*/*BNIP3* (**F**) mRNA expression (high/high, high/low, low/high, and low/low groups, respectively).

Next, we interrogated the TCGA database to assess the prognostic value of a molecular signature that includes glycolysis (*HK2*, *PFKM*, and *PKM*), autophagy (*MAP1LC3B*), and mitophagy (*BNIP3*)-associated markers (Figure 6). We correlated the selected genes based on the level of co-expression, and AML patients were subdivided into four groups, namely high/high, high/low, low/high, and low/low, respectively. All the stratification signatures with low glycolysis and high autophagy–mitophagy correlated with a better clinical outcome for AML patients, and the most significant difference was observed with *PFKM*/*MAP1LC3B* (*p* = 0.0004) and *PFKM*/*BNIP3* (*p* = 0.0030) signatures (Figure 6C,D), followed by *PKM*/*MAP1LC3B* and *PKM*/*BNIP3* (Figure 6E,F) and *HK2*/*MAP1LC3B* and *HK2*/*BNIP3* (Figure 6A,B), reflecting the trend in individual prognostic analysis described above. Overall, these findings indicate that the glycolysis/autophagy–mitophagy axis represents a valid prognostic signature for AML.

**Figure 7:**
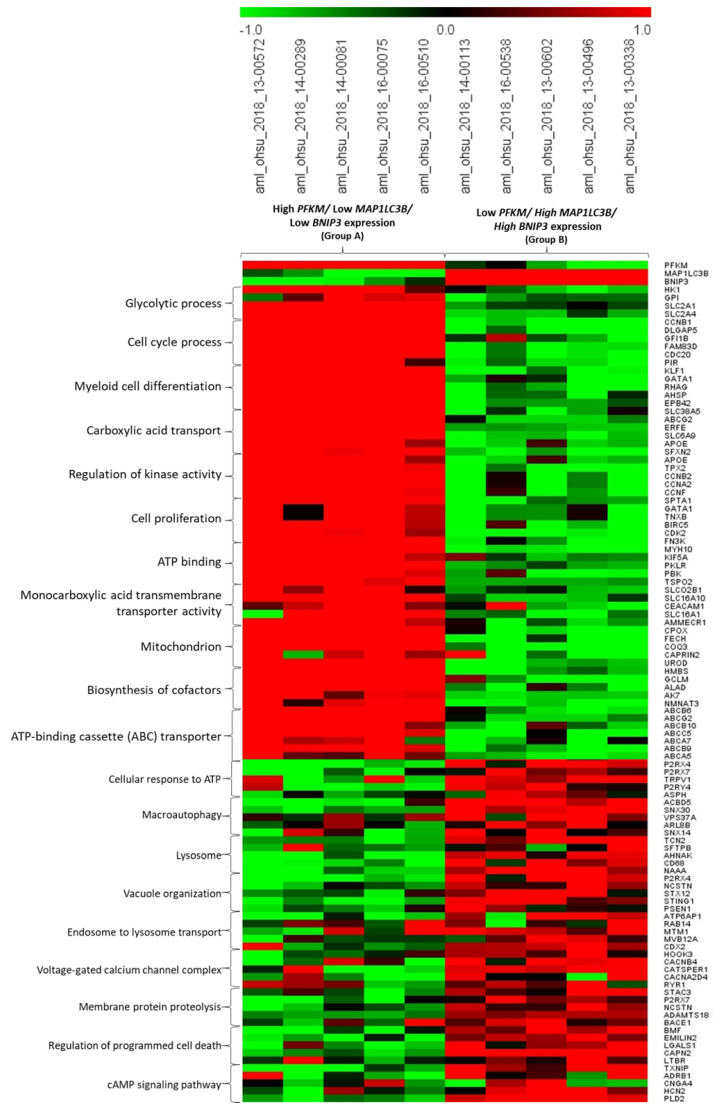
Comparison of differentially expressed genes in two groups of patients stratified based on *PFKM*, *MAP1LC3B*, and *BNIP3* expression. Patients were in Group A (characterized by high *PFKM*/ low *MAP1LC3B*/low *BNIP3* expression) and Group B (characterized by low *PFKM*/high *MAP1LC3B*/high *BNIP3* expression). The heatmap includes the top five significant genes related to each biological process differentially correlated to *PFKM* expression.

In the subsequent analysis, AML patients were divided into two groups based on the opposite expression of *PFKM*, *MAP1LC3B*, and *BNIP3*. We selected five patients for each group as follows: (i) Group A included patients with high *PFKM*/low *MAP1LC3B*/low *BNIP3* expression (i.e., high glycolysis and low autophagy–mitophagy), whereas (ii) Group B included those with low *PFKM*/high *MAP1LC3B*/high *BNIP3* expression (i.e., low glycolysis and high autophagy–mitophagy). The most significant differentially expressed genes (DEGs) were screened and selected for the main biological process reported in Figure 3. The heatmap shown in Figure 7 emphasizes that AML patients in Group A (showing high *PFKM*/low *MAP1LC3B*/low *BNIP3* expression and poor prognosis) were characterized by the upregulation of a range of transcripts involved in oncogenic pathways, including glucose transporters (*SLC2A1*, *SLC2A4*), lactate/pyruvate transporters (*SLC16A1*), amino acids transporters (*SLC6A9*, *SLC15A10*), positive cell cycle regulation (*CCNB1*, *CCNB2*, *CCNA2*), and the biosynthesis of cofactors (*NMNAT3*, *GCLM*, *HMBS*). In contrast, AML patients of Group B (showing low *PFKM*/high *MAP1LC3B*/high *BNIP3* expression and better prognosis) showed a marked downregulation of genes crucial for myeloid cell differentiation and homeostasis (*KLF1*, *GATA1*, *RHAG*, *AHSP*, *EPBA2*, *MYH10*), multidrug efflux transporters (*ABCA5*, *ABCA7*, *ABCB6*, *ABCB9*, *ABCB10*, *ABCC5*, *ABCG2*), and apoptosis inhibition (*BIRC5C*), together with an upregulation of a wide range of transcripts involved in autophagy/lysosomal trafficking and proteolysis (*ACBD5*, *SNX30*, *VPS37A*, *ARL8B*, *RAB14*, *ATP16A1*), mitophagy (*SNX14*), beta-interferon production (STING1), and the positive regulation of programmed cell death (*EMILIN2*, *CAPN2*, *LTBR*). Taken together, this data reinforces the view that a low glycolysis/high autophagy-mitophagy signature can predict a favorable clinical outcome for AML patients.

## 3. Discussion

Autophagy is a lysosomal-driven catabolic process that facilitates the metabolic adaptation of cancer cells by maintaining basal levels of metabolites and biosynthetic intermediates [20]. Autophagy acts at a crossroad between cancer cell survival and cell death pathways, thus supporting either chemoresistance or onco-suppressive functions [21]. Autophagy plays a pivotal role in hematopoietic stem cell homeostasis, and it is dysregulated in blood tumors [22,23].

AML is often charged with chemoresistance and high recurrence rates, traits likely driven by acquired metabolic reprogramming [24].

In particular, aerobic glycolysis (the Warburg effect) provides the metabolic intermediates and energy needed for cancer cell proliferation and supports leukemic cell plasticity, therapy resistance, and disease recurrence in AML [4,25]. Glycolysis goes in parallel with dysfunctional mitochondria, which must be cleared by mitophagy, the targeted autophagy degradation of mitochondria [26].

Pre-clinical studies have shown that lack of sequestosome 1 (SQSTM1/P62), a scaffold protein that sequesters within the autophagosome the substrates to be degraded, associates with the accumulation of damaged mitochondria which in turn negatively impacts the survival of leukemic cells [27]. Similarly, the mitophagy inhibitor XRK3F2 targeting P62 was found to counteract the tumorigenic potential of leukemia-initiating cells both in mice and patient-derived tumor xenograft AML models [28]. Moreover, elevated levels of the mitophagy marker PTEN-induced kinase 1 (PINK1) enable leukemic cells to mend impaired mitochondria, evade apoptosis, and persist in proliferation despite mitochondrial dysfunction [29].

The above data support the individual impact of altered glucose metabolism and of dysfunctional autophagy–mitophagy in AML cells, yet a consolidated overview of interrelated network between these processes has yet to be decrypted in AML.

In the present work, we investigated the prognostic role of the combined glycolysis/autophagy–mitophagy signature in AML. We found that a high expression of glycolytic markers *HK2*, *PFKM*, and *PKM* correlates with a shorter overall survival rate. From our analysis, *PFKM* (which encodes for phosphofructokinase, muscle isoform), one of the most important regulatory enzymes that catalyze the rate-limiting step of glycolysis, emerges as a robust prognostic factor for the stratification of AML in good or poor responders. Accordingly, it was found that *PFKM* was expressed at low level in AML patients categorized in the favorable-risk group. This observation is further supported by evidence that the upregulation of *PFKP* (phosphofructokinase, platelet isoform) is associated with disease progression and worse clinical outcomes of AML patients [30,31].

The transcriptomic analysis performed on TCGA patients’ cohort revealed that *PFKM* expression is positively associated with genes regulating pro-survival pathways, such as myeloid cell maturation, the regulation of DNA replication and cell cycle progression, the biosynthesis of essential cofactors, and cellular energy metabolism. On the other side, *PFKM* negatively correlates with genes associated with mitophagy, autophagosome formation, lysosomal proteolysis, and apoptosis regulation.

We hypothesized that low glucose metabolism together with an upregulation of the autophagy–mitophagy axis may confer a favorable prognosis for AML patients. Multiple-variable survival analysis in fact shows that a low expression of glycolytic markers (particularly, *HK2*, *PFKM*, and *PKM*) together with active autophagy–mitophagy (high *MAP1LC3B* and high *BNIP3*) predispose AML patients to better prognosis, which could be related to the sensitization of cancer cells to therapy via BECLIN-1-dependent autophagy–mitophagy.

Remarkably, from the transcriptomic analysis performed on AML patients (stratified based on *PFKM*/*MAP1LCB*/*BNIP3* expression) emerges that those with low glycolysis and active autophagy–mitophagy (displaying a better prognosis) exhibit a downregulation of a subset of genes required for the correct maturation and proliferation of myeloid cells, lactate/pyruvate transporters, multidrug efflux transporters, and evasion from programmed cell death. In particular, we identified *MYH10* (Myosin Heavy Chain 10), *SLC16A1* (Solute carrier family 16 member 1), ATP-binding cassette transporters, and *BIRC5C* (Baculoviral IAP Repeat Containing 5C).

MYH10 is a protein that prevents megakaryocyte ploidization and maturation and promotes the migration of leukemia cells. Accordingly, AML patients displaying low *MYH10* expression are associated with a more favorable prognosis [32].

*SLC16A1* encodes for monocarboxylate transporter 1 (MCT1), a lactate transporter that contributes to shaping an acidic tumor microenvironment. Targeting MCT1 has been reported to effectively sensitize AML cells to BET inhibitors both in vitro and in vivo [33].

*BIRC5C* encodes for Survivin, a protein that has been found to be overexpressed in 60% of adult AML patient samples. BIRC5C mediates the inhibition of apoptosis and supports cancer cell proliferation, conferring a worse clinical outcome to AML patients [34].

Several ATP-binding cassette transporters identified in the transcriptomic analysis have been reported to associate with the multidrug resistance profile of AML patients [35], representing a major challenge for overcoming the low efficacy of therapeutic strategies.

Interestingly, the same group of patients displayed an upregulation of *STING1* (stimulator of interferon response cGAMP interactor 1), which induces beta-interferon production. This finding holds potent translational relevance, given that agonists of the stimulation of interferon response have been considered a valid option for combination therapy in AML patients to reduce the impact of minimal residual disease upon relapse [36,37].

Collectively, these findings suggest that the strategic utilization of autophagy–mitophagy signatures, in conjunction with metabolic modifiers, holds the potential to accurately predict favorable clinical outcomes for AML patients.

In conclusion, our data support the view that autophagy–mitophagy induction along with glucose restriction is beneficial for AML patients, suggesting that autophagy inducers (like caloric restriction mimetics, for instance) together with glucose restriction mimetics may represent a valid adjuvant strategy for overcoming therapy resistance while ameliorating clinical outcomes.

## 4. Materials and Methods

### 4.1. TCGA AML Database

All clinical data and gene expression profiles were retrieved from the TCGA database (www.cBioportal.org, last accessed on 10 September 2024). The acute myeloid leukemia (AML) dataset accounts for 622 patients (OHSU, Nature 2018). However, RNA-seq and whole clinical data are available for only 405 patients. The AML cohort includes 235 males and 170 females, and the median age was 61 years (range: 2–87). Information regarding the therapy administered to the patients was mentioned on the TCGA portal: 372 patients were undergoing chemotherapy while 33 were not. The patients were on standard chemotherapy along with target therapy or bone marrow transplants.

### 4.2. Screening of the DEGs in Correlation with PKFM

RNA-seq data was retrieved from 405 AML patients of the TCGA repository. Pearson’s and Spearman’s correlation analyses were performed to identify the correlation and differentially expressed genes (DEGs). Pearson’s correlation analysis revealed a significant relationship between *PFKM* and other genes (i.e., 18,251 genes). Regression was estimated using Pearson’s correlation coefficients (r) and relative *p*-values. TBtool (https://github.com/CJ-Chen/TBtools/, accessed on 25 August 2024) was used to identify the DEGs. Cut-off criteria were based on Spearman’s correlation value, i.e., a correlation coefficient value higher than +0.30 (positively correlated) or lower than −0.30 (negatively correlated), and a *p*-value < 0.001 (−log10 (*p*-value)) threshold was fixed above 2.0.

### 4.3. Gene Ontology and Pathway Enrichment Analysis of DEGs

The enrichment analysis of DEGs was performed using the DAVID bioinformatics functional annotation tool (https://david.ncifcrf.gov/summary.jsp, accessed on 30 August 2024) to obtain Gene Ontology (GO) biological processes and Kyoto Encyclopedia of Genes and Genomes (KEGG) pathways. Data are represented as a bar graph displaying the number of transcripts belonging to each positively and negatively correlated biological process. MeV4 (https://webmev.tm4.org/, accessed on 10 September 2024) was utilized to create heat maps based on differential expression values.

### 4.4. Statistical Analysis of Gene Expression and Clinical Outcomes

Survival curves and correlation analysis results were obtained by retrieving data from the TCGA cohort of AML (N = 405) patients. The *HK2*, *PFKM*, *PKM*, *BECN1*, *MAP1LC3B*, *PRKN*, *BNIP3*, and *BNIP3L* mRNA expression levels were sub-categorized into high- and low-mRNA-expression groups based on z-score values. Low versus high mRNA expression was defined relative to the median expression level. The correlation between the mRNA expression of these selected genes was represented as a box plot.

To reduce the potential bias from dichotomization, mRNA expression levels were compared considering high- and low-expression-based groups using a *t*-test (Welch’s two-sample *t*-test). All cut-off values were set before the analysis, and all the tests were two-tailed. Overall survival curves were estimated using the Kaplan–Meier method and the log-rank test to evaluate the difference in the distribution of survival curves. Univariate and multivariable survival analyses were studied using SAS software (v.9.4). To reduce to the minimum the risk for bias in multiple-variable analysis, comparisons were performed following the Cox regression model. The log-rank test was used to determine the statistical significance. A *p*-value < 0.05 was considered significant. All statistical analyses were performed using R (3.6.1 version, The R Foundation for Statistical Computing, Vienna, Austria) and SAS software (9.4. version, SAS Institute Inc., Cary, NC, USA).

## Data Availability

The original contributions presented in the study are included in the article/Supplementary Material, further inquiries can be directed to the corresponding authors.

## Supplementary Materials

The following supporting information can be downloaded at https://www.mdpi.com/article/10.3390/ijms252111527/s1.

## References

[1] Papaemmanuil E., Gerstung M., Bullinger L., Gaidzik V.I., Paschka P., Roberts N.D., Potter N.E., Heuser M., Thol F., Bolli N. (2016). Genomic Classification and Prognosis in Acute Myeloid Leukemia. N. Engl. J. Med..

[2] Roman Diaz J.L., Vazquez Martinez M., Khimani F. (2024). New Approaches for the Treatment of AML beyond the 7+3 Regimen: Current Concepts and New Approaches. Cancers.

[3] Panina S.B., Pei J., Kirienko N.V. (2021). Mitochondrial Metabolism as a Target for Acute Myeloid Leukemia Treatment. Cancer Metab..

[4] Zhang J., Gu Y., Chen B. (2019). Mechanisms of Drug Resistance in Acute Myeloid Leukemia. Onco Targets Ther..

[5] Yang Y., Pu J., Yang Y. (2024). Glycolysis and Chemoresistance in Acute Myeloid Leukemia. Heliyon.

[6] Mathew M., Nguyen N., Bhutia Y., Sivaprakasam S., Ganapathy V. (2024). Metabolic Signature of Warburg Effect in Cancer: An Effective and Obligatory Interplay between Nutrient Transporters and Catabolic/Anabolic Pathways to Promote Tumor Growth. Cancers.

[7] Ju H.-Q., Zhan G., Huang A., Sun Y., Wen S., Yang J., Lu W.-H., Xu R.-H., Li J., Li Y. (2017). ITD Mutation in FLT3 Tyrosine Kinase Promotes Warburg Effect and Renders Therapeutic Sensitivity to Glycolytic Inhibition. Leukemia.

[8] Debnath J., Gammoh N., Ryan K.M. (2023). Autophagy and Autophagy-Related Pathways in Cancer. Nat. Rev. Mol. Cell Biol..

[9] Tan V.P., Miyamoto S. (2015). HK2/Hexokinase-II Integrates Glycolysis and Autophagy to Confer Cellular Protection. Autophagy.

[10] Gao Y., Wu Z., Chen Y., Shang G., Zeng Y., Gao Y. (2023). A Global Bibliometric and Visualized Analysis of the Links between the Autophagy and Acute Myeloid Leukemia. Front. Pharmacol..

[11] Mortensen M., Watson A.S., Simon A.K. (2011). Lack of Autophagy in the Hematopoietic System Leads to Loss of Hematopoietic Stem Cell Function and Dysregulated Myeloid Proliferation. Autophagy.

[12] Seo W., Silwal P., Song I.-C., Jo E.-K. (2022). The Dual Role of Autophagy in Acute Myeloid Leukemia. J. Hematol. Oncol..

[13] Piya S., Kornblau S.M., Ruvolo V.R., Mu H., Ruvolo P.P., McQueen T., Davis R.E., Hail N., Kantarjian H., Andreeff M. (2016). Atg7 Suppression Enhances Chemotherapeutic Agent Sensitivity and Overcomes Stroma-Mediated Chemoresistance in Acute Myeloid Leukemia. Blood.

[14] Bosnjak M., Ristic B., Arsikin K., Mircic A., Suzin-Zivkovic V., Perovic V., Bogdanovic A., Paunovic V., Markovic I., Bumbasirevic V. (2014). Inhibition of MTOR-Dependent Autophagy Sensitizes Leukemic Cells to Cytarabine-Induced Apoptotic Death. PLoS ONE.

[15] Nombela-Arrieta C., Pivarnik G., Winkel B., Canty K.J., Harley B., Mahoney J.E., Park S.-Y., Lu J., Protopopov A., Silberstein L.E. (2013). Quantitative Imaging of Haematopoietic Stem and Progenitor Cell Localization and Hypoxic Status in the Bone Marrow Microenvironment. Nat. Cell Biol..

[16] Parmar K., Mauch P., Vergilio J.-A., Sackstein R., Down J.D. (2007). Distribution of Hematopoietic Stem Cells in the Bone Marrow According to Regional Hypoxia. Proc. Natl. Acad. Sci. USA.

[17] Bruno S., Mancini M., De Santis S., Monaldi C., Cavo M., Soverini S. (2021). The Role of Hypoxic Bone Marrow Microenvironment in Acute Myeloid Leukemia and Future Therapeutic Opportunities. Int. J. Mol. Sci..

[18] Wu J., Niu J., Li X., Li Y., Wang X., Lin J., Zhang F. (2014). Hypoxia Induces Autophagy of Bone Marrow-Derived Mesenchymal Stem Cells via Activation of ERK1/2. Cell Physiol. Biochem..

[19] Dykstra K.M., Fay H.R.S., Massey A.C., Yang N., Johnson M., Portwood S., Guzman M.L., Wang E.S. (2021). Inhibiting Autophagy Targets Human Leukemic Stem Cells and Hypoxic AML Blasts by Disrupting Mitochondrial Homeostasis. Blood Adv..

[20] Goldsmith J., Levine B., Debnath J. (2014). Autophagy and Cancer Metabolism. Methods Enzymol..

[21] Nencioni A., Cea M., Montecucco F., Longo V.D., Patrone F., Carella A.M., Holyoake T.L., Helgason G.V. (2013). Autophagy in Blood Cancers: Biological Role and Therapeutic Implications. Haematologica.

[22] Rothe K., Porter V., Jiang X. (2019). Current Outlook on Autophagy in Human Leukemia: Foe in Cancer Stem Cells and Drug Resistance, Friend in New Therapeutic Interventions. Int. J. Mol. Sci..

[23] Stergiou I.E., Kapsogeorgou E.K. (2021). Autophagy and Metabolism in Normal and Malignant Hematopoiesis. Int. J. Mol. Sci..

[24] Kreitz J., Schönfeld C., Seibert M., Stolp V., Alshamleh I., Oellerich T., Steffen B., Schwalbe H., Schnütgen F., Kurrle N. (2019). Metabolic Plasticity of Acute Myeloid Leukemia. Cells.

[25] Liberti M.V., Locasale J.W. (2016). The Warburg Effect: How Does It Benefit Cancer Cells?. Trends Biochem. Sci..

[26] Song C., Pan S., Zhang J., Li N., Geng Q. (2022). Mitophagy: A Novel Perspective for Insighting into Cancer and Cancer Treatment. Cell Prolif..

[27] Nguyen T.D., Shaid S., Vakhrusheva O., Koschade S.E., Klann K., Thölken M., Baker F., Zhang J., Oellerich T., Sürün D. (2019). Loss of the Selective Autophagy Receptor P62 Impairs Murine Myeloid Leukemia Progression and Mitophagy. Blood.

[28] Li Y., Li Y., Yin J., Wang C., Yang M., Gu J., He M., Xu H., Fu W., Zhang W. (2021). A Mitophagy Inhibitor Targeting P62 Attenuates the Leukemia-Initiation Potential of Acute Myeloid Leukemia Cells. Cancer Lett..

[29] Xu Y., Tran L., Tang J., Nguyen V., Sewell E., Xiao J., Hino C., Wasnik S., Francis-Boyle O.L., Zhang K.K. (2022). FBP1-Altered Carbohydrate Metabolism Reduces Leukemic Viability through Activating P53 and Modulating the Mitochondrial Quality Control System In Vitro. Int. J. Mol. Sci..

[30] Luo X., Zheng D., Zheng R., Wang C., Xu L., Tan H. (2018). The Platelet Isoform of Phosphofructokinase in Acute Myeloid Leukemia: Clinical Relevance and Prognostic Implication. Blood.

[31] Peng J., Li P., Li Y., Quan J., Yao Y., Duan J., Liu X., Li H., Yuan D., Wang X. (2023). PFKP Is a Prospective Prognostic, Diagnostic, Immunological and Drug Sensitivity Predictor across Pan-Cancer. Sci. Rep..

[32] Wei Y., Lu W., Yu Y., Zhai Y., Guo H., Yang S., Zhao C., Zhang Y., Liu J., Liu Y. (2021). MiR-29c&b2 Encourage Extramedullary Infiltration Resulting in the Poor Prognosis of Acute Myeloid Leukemia. Oncogene.

[33] Boët E., Sarry J.-E. (2024). Targeting Metabolic Dependencies Fueling the TCA Cycle to Circumvent Therapy Resistance in Acute Myeloid Leukemia. Cancer Res..

[34] Greiner J., Brown E., Bullinger L., Hills R.K., Morris V., Döhner H., Mills K.I., Guinn B. (2021). Survivin’ Acute Myeloid Leukaemia—A Personalised Target for Inv(16) Patients. Int. J. Mol. Sci..

[35] Vasconcelos F.C., De Souza P.S., Hancio T., De Faria F.C.C., Maia R.C. (2021). Update on Drug Transporter Proteins in Acute Myeloid Leukemia: Pathological Implication and Clinical Setting. Crit. Rev. Oncol./Hematol..

[36] Song X., Peng Y., Wang X., Chen Q., Lan X., Shi F. (2022). The Stimulator of Interferon Genes (STING) Agonists for Treating Acute Myeloid Leukemia (AML): Current Knowledge and Future Outlook. Clin. Transl. Oncol..

[37] Healy F.M., Dahal L.N., Jones J.R.E., Floisand Y., Woolley J.F. (2021). Recent Progress in Interferon Therapy for Myeloid Malignancies. Front. Oncol..

