# Supplementary material for: High Mitophagy and Low Glycolysis Predict Better Clinical Outcomes in Acute Myeloid Leukemias
Source: Int J Mol Sci. 2024 Oct 27;25(21):11527. doi: 10.3390/ijms252111527 (PMC11546612; doi:10.3390/ijms252111527)
Supplement: Supplementary file 1 [file ijms-25-11527-s001.zip › ijms-3244755-supplementary.pdf]

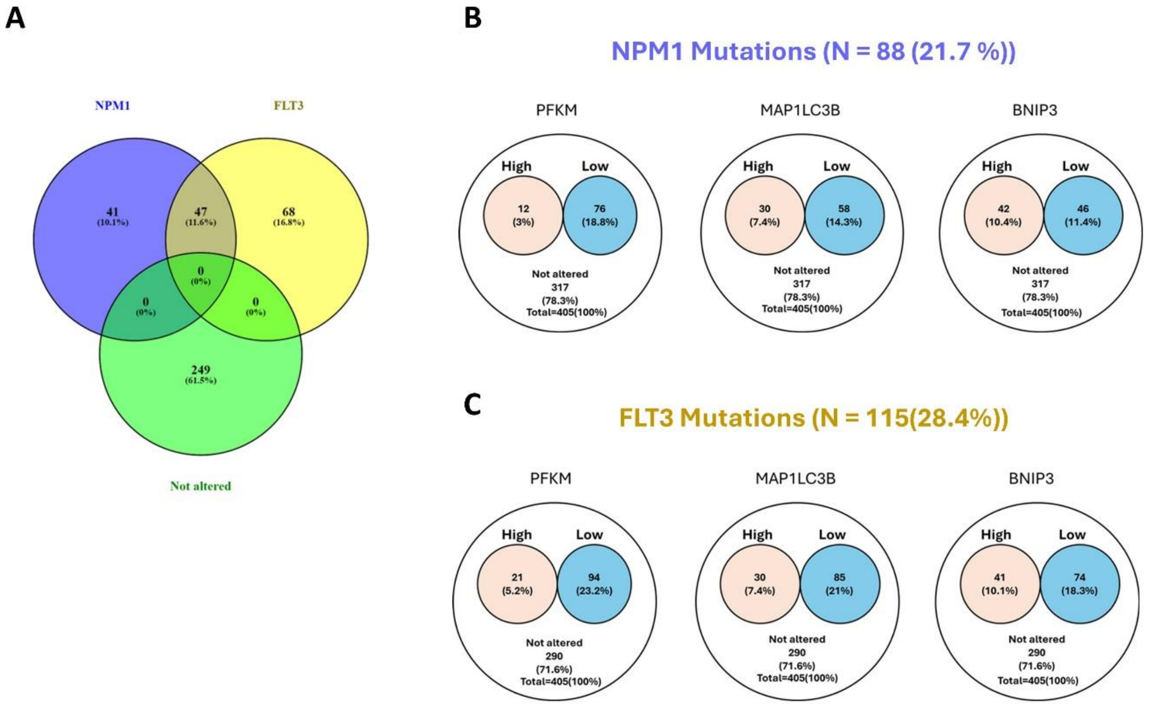

Supplementary Figure S1. **Correlation between glycolytic markers/autophagy-mitophagy and cytogenetic/molecular alterations.** A) Venn diagram showing the percentage of genetic/molecular alterations in AML patients' datasets (TCGA, OHSU, Nature 2018). B) Venn diagram showing the distribution of *PFKM*, *MAP1LC3B*, and *BNIP3* mRNA expression levels based on *NPM1* mutational status. C) Box plots showing the distribution of *PFKM*, *MAP1LC3B*, and *BNIP3* mRNA expression levels based on *FLT3* mutational status.

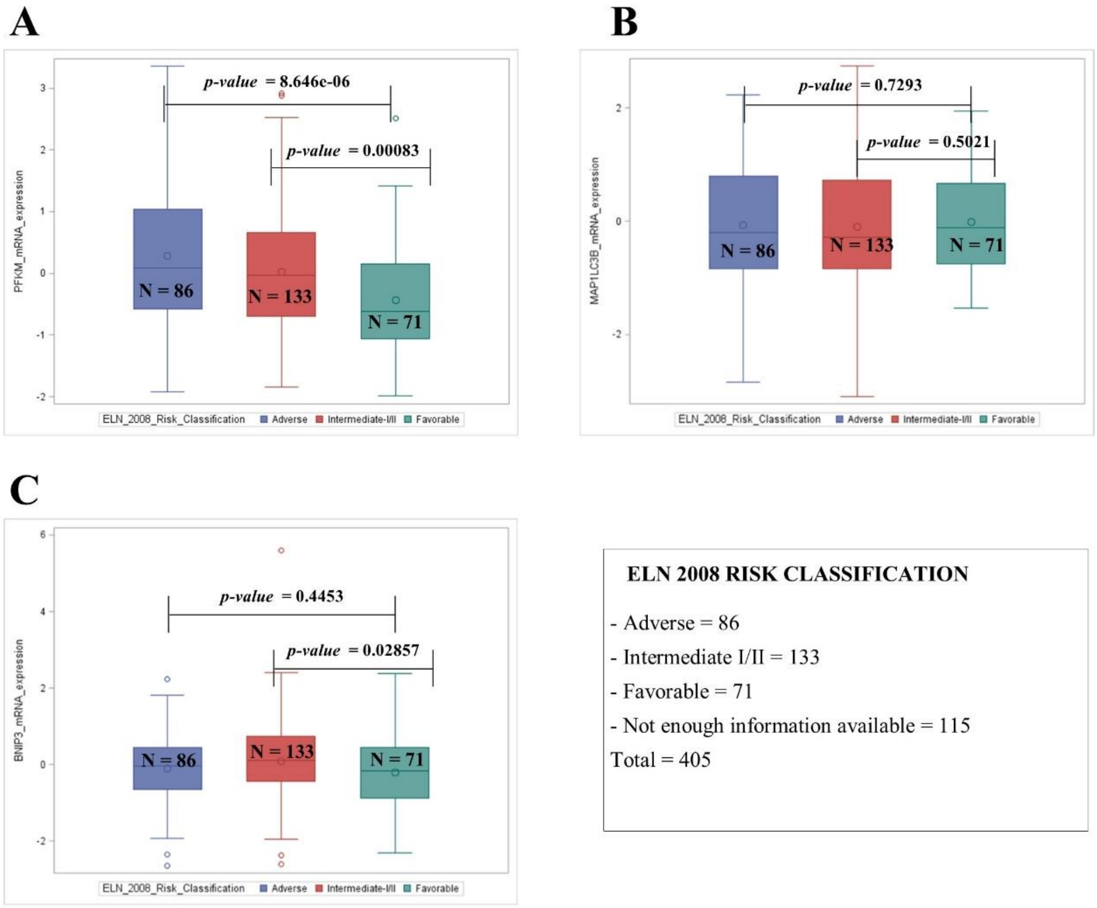

Supplementary Figure S2. **Correlation between ELN 2008 risk classification and glycolytic markers/autophagy-mitophagy.** Box plots showing the distribution of *PFKM* (A), *MAP1LC3B* (B), and *BNIP3* (C) mRNA expression levels based on risk score.

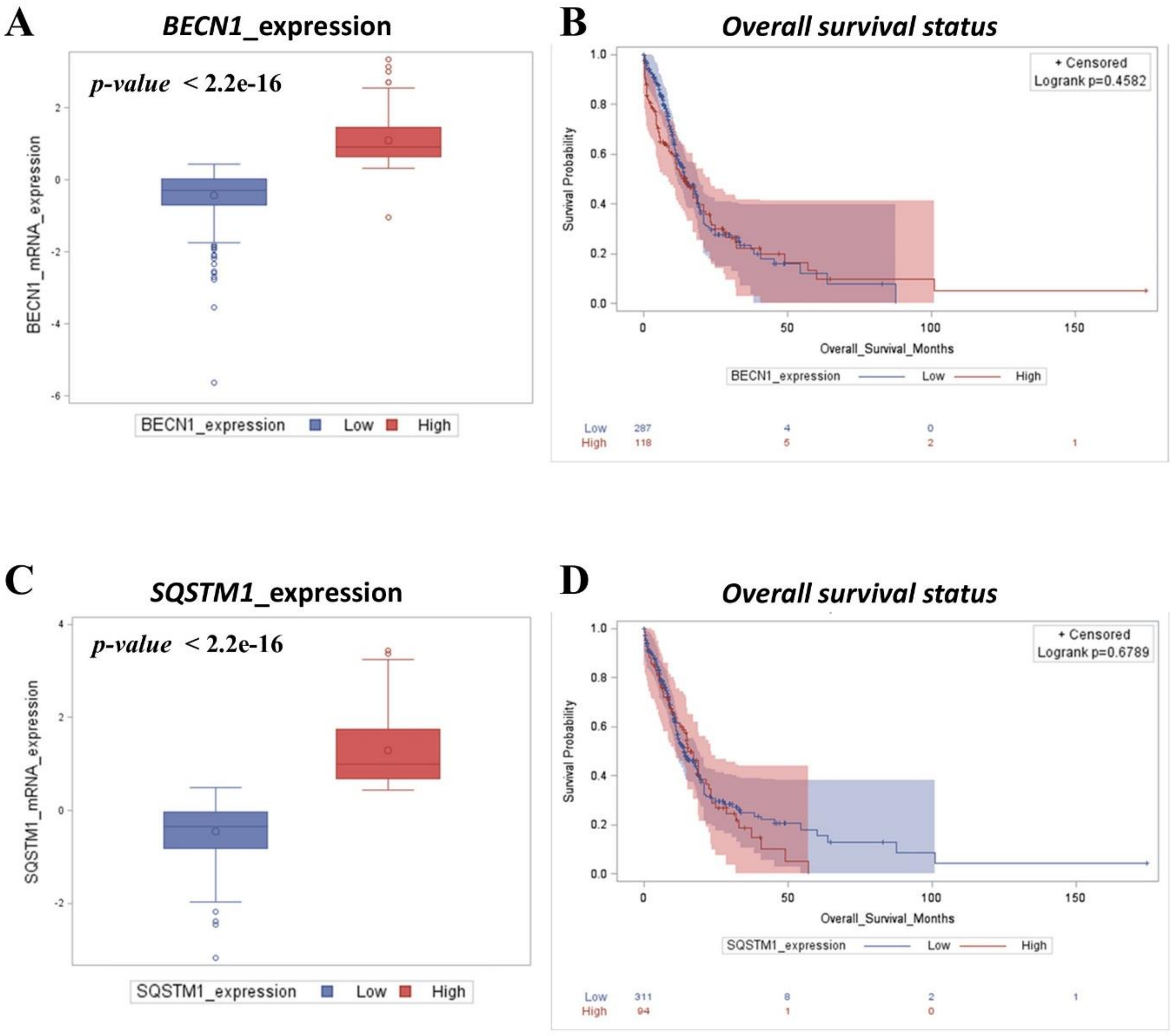

Supplementary Figure S3. High expression of *BECN1* and low expression of *SQSTM1* are associated with good prognosis in AML patients. A-C) Box plots showing the distribution of *BECN1* (A), and *SQSTM1* (C) mRNA expression levels in AML patients (high vs. low). B-D) Kaplan-Meier curves depicting the overall survival rate of AML patients, respectively, based on differential *BECN1* (B), and *SQSTM1* (D) mRNA expression levels (low vs. high).

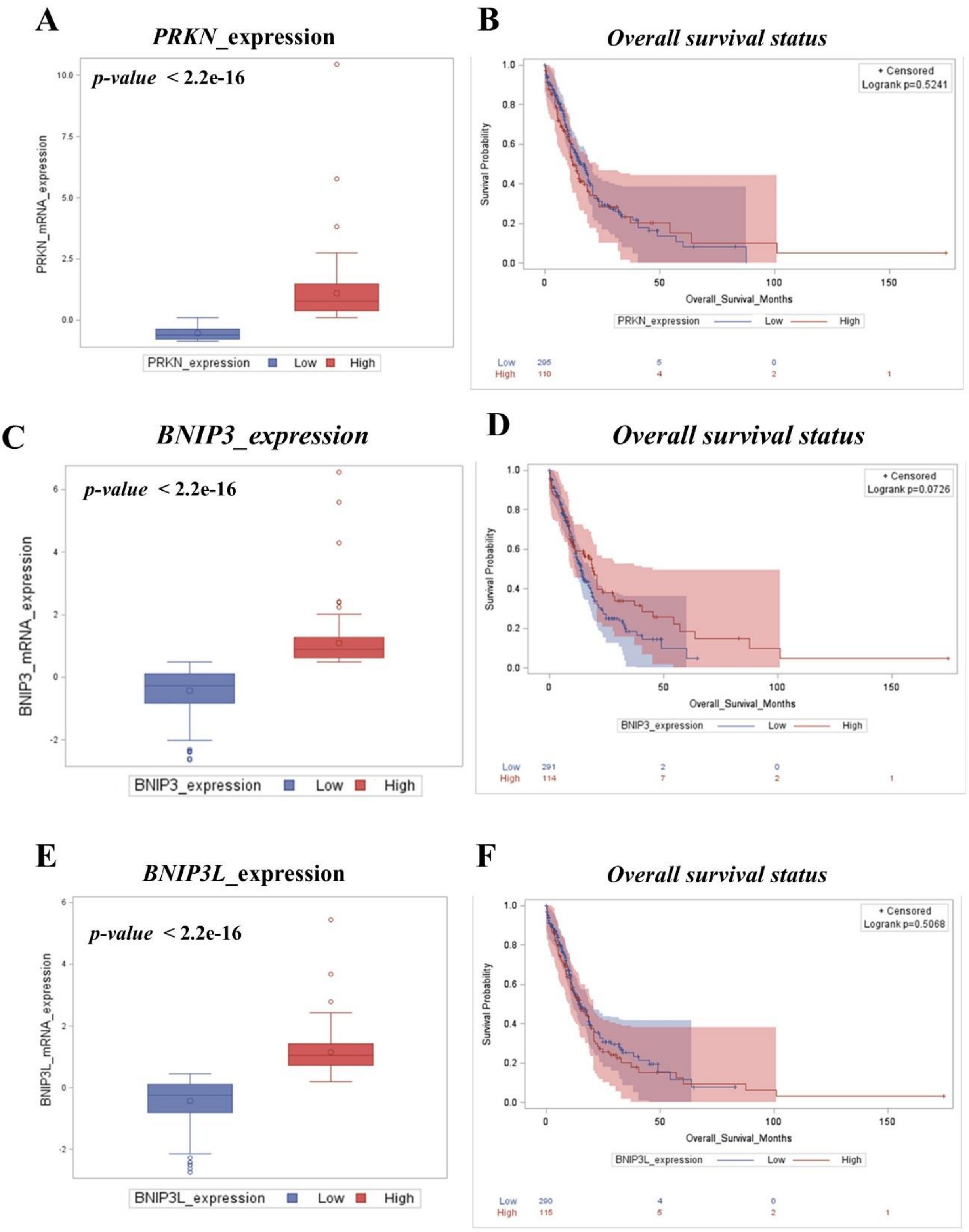

Supplementary Figure S4. High expression of *PRKN*, *BNIP3*, and *BNIP3L* is associated with good prognosis in AML patients. A-C-E) Box plots showing the distribution of *PRKN* (A), *BNIP3* (C), and *BNIP3L* (E) mRNA expression levels in AML patients (high vs. low). B-D-F) Kaplan-Meier curves depicting the overall survival rate of AML patients, respectively, based on differential *PRKN* (B), *BNIP3* (D), and *BNIP3L* (F) mRNA expression levels (low vs. high).
